# 

**Published:** 1892-11

**Authors:** 


ESTABLISHED 1855. SUBSCRIPTION, $2 00.

### ATLANTA MEDICAL AND SURGICAL JOURNAL.

OLD SERIES, VOL. XXVI. NEW SERIES, VOL. IX. } ATLANTA, GA., NOVEMBER, 1892. No. 9.

LUTHER B. GRANDY, M. D., MANAGING EDITOR. M. B. HUTCHINS, M. D. BUSINESS MANAGER.

